# Interactive Effects of Elevated [CO_2_] and Water Stress on Physiological Traits and Gene Expression during Vegetative Growth in Four Durum Wheat Genotypes

**DOI:** 10.3389/fpls.2016.01738

**Published:** 2016-11-22

**Authors:** Susan Medina, Rubén Vicente, Amaya Amador, José Luis Araus

**Affiliations:** ^1^Integrative Crop Ecophysiology Group, Plant Physiology Section, Faculty of Biology, University of BarcelonaBarcelona, Spain; ^2^Crop Physiology Laboratory, International Crops Research Institute for Semi-Arid TropicsPatancheru, India; ^3^Unitat de Genòmica, Centres Científics i Tecnològics, Universitat de BarcelonaBarcelona, Spain

**Keywords:** climate change, durum wheat, elevated [CO_2_], genotypic variability, stable isotopes, transcript levels, vegetative growth, water stress

## Abstract

The interaction of elevated [CO_2_] and water stress will have an effect on the adaptation of durum wheat to future climate scenarios. For the Mediterranean basin these scenarios include the rising occurrence of water stress during the first part of the crop cycle. In this study, we evaluated the interactive effects of elevated [CO_2_] and moderate to severe water stress during the first part of the growth cycle on physiological traits and gene expression in four modern durum wheat genotypes. Physiological data showed that elevated [CO_2_] promoted plant growth but reduced N content. This was related to a down-regulation of Rubisco and N assimilation genes and up-regulation of genes that take part in C-N remobilization, which might suggest a higher N efficiency. Water restriction limited the stimulation of plant biomass under elevated [CO_2_], especially at severe water stress, while stomatal conductance and carbon isotope signature revealed a water saving strategy. Transcript profiles under water stress suggested an inhibition of primary C fixation and N assimilation. Nevertheless, the interactive effects of elevated [CO_2_] and water stress depended on the genotype and the severity of the water stress, especially for the expression of drought stress-responsive genes such as dehydrins, catalase, and superoxide dismutase. The network analysis of physiological traits and transcript levels showed coordinated shifts between both categories of parameters and between C and N metabolism at the transcript level, indicating potential genes and traits that could be used as markers for early vigor in durum wheat under future climate change scenarios. Overall the results showed that greater plant growth was linked to an increase in N content and expression of N metabolism-related genes and down-regulation of genes related to the antioxidant system. The combination of elevated [CO_2_] and severe water stress was highly dependent on the genotypic variability, suggesting specific genotypic adaptation strategies to environmental conditions.

## Introduction

Food security is facing new challenges nowadays due to the increase in the world population and the impacts of climate change on agriculture and food supply. Wheat is a very important crop for the human diet, ranking in fourth position in terms of the world's most important crops by production quantity after sugarcane, maize, and rice (FAO, 2013). Although bread wheat dominates global wheat production, durum wheat is an economically and culturally important staple crop in the Mediterranean region, used for the production of pasta, bread, burghul, couscous, and freekeh (Habash et al., 2009). In the second half of the twentieth century, local durum wheat landraces were replaced by improved semi-dwarf cultivars, which showed higher yield and harvest index (Soriano et al., 2016). In the early 1970s, introduction of germplasm from CIMMYT (International Maize and Wheat Improvement Centre) increased grain yield (Sanchez-Garcia et al., 2013). Improvement in wheat yield per unit area constitutes one of the largest challenges to be addressed by breeding programs, covering numerous research areas (McKersie, 2015). Projections of wheat production assume that the growth rate will be lower than the historical growth rates reported in the second half of the twentieth century (Bort et al., 2014; Nakhforoosh et al., 2015), with insignificantly higher yields in modern wheat genotypes released in recent years (Sanchez-Garcia et al., 2013). It is unlikely that any improvements will support the increase in world population or mitigate against future extreme weather events (Araus et al., 2002; Alexandratos and Bruinsma, 2012; Trnka et al., 2014).

Observations of the climate system confirm that Earth's mean surface temperature is increasing rapidly as a consequence of the anthropogenic emissions of CO_2_ and other greenhouse gases (IPCC, 2013). The atmospheric concentration of CO_2_ ([CO_2_]) has increased by more than 40% since the beginning of the industrial revolution and is expected to double by the end of this century (IPCC, 2013). As atmospheric [CO_2_] is currently a limiting factor for C_3_ photosynthesis, the primary effect of a short-term exposure to elevated [CO_2_] includes an initial stimulation of photosynthesis due to both enrichment of substrate for ribulose bisphosphate carboxylase oxygenase (Rubisco) carboxylation and inhibition of competitive Rubisco oxygenation which may eventually contribute to a higher biomass (Stitt and Krapp, 1999; Long et al., 2006). High [CO_2_] also induces a stomatal closure leading to a better leaf water status. However, growth over the long-term under elevated [CO_2_] leads to a down-regulation of photosynthetic capacity, which has been related to a decline in Rubisco protein content and activity, together with a higher carbohydrate accumulation and a decline in N concentration and protein content in wheat (Aranjuelo et al., 2011, 2013; Vicente et al., 2015a,b). This phenomenon suggests that regulatory mechanisms may occur in the plant, e.g., end-product inhibition, carbon sink limitation, biomass dilution effects, or a decline in nutrient uptake and/or assimilation (Stitt and Krapp, 1999; Vicente et al., 2015a). Moreover, elevated [CO_2_] leads to an altered expression pattern of genes involved in the photosynthetic apparatus, the distribution of C, respiration, and N metabolism in durum wheat (Vicente et al., 2015b).

Increasing greenhouse gas emissions may cause further warming together with rainfall reduction in the next decades, which will increase the frequency and intensity of drought in the Mediterranean basin (Habash et al., 2009; IPCC, 2013; McKersie, 2015). For the Iberian Peninsula it is predicted that drought stress can occur at any growth stage of wheat (Russo et al., 2015), with the grain-filling phase being the most studied. However, the number of studies focusing on drought stress during early growth is limited. Although rainfall has been traditionally most abundant and evapotranspiration the lowest during winter, the occurrence of drought in winter months during the early stages of the crop cycle has been reported in recent times (Russo et al., 2015). This can further constrain wheat growth and thus final grain yield, mostly through a decrease in the ear density and number of kernels per unit crop area (Araus et al., 2008; Rebolledo et al., 2013). In addition, a constitutive (i.e., in absence of water stress) rapid development of wheat plants (early vigor) could be a positive trait and relevant for further avoiding drought stress-related consequences at both early and late growth stages. Early vigor could benefit plant growth and yield by increasing resource acquisition, shading the soil, preventing evaporation from it, and suppressing weeds (Maydup et al., 2012; Bort et al., 2014; Pang et al., 2014). As a consequence, differences in early growth (tillering and further stem elongation) will affect the number of fertile stems (and thus the ear density) and the size of the ears (and thus the potential number of grains per ear), which are the main contributors determining grain yield (Guo et al., 2016).

Plant responses to water stress define a complex and sophisticated regulatory network comprising physiological, biochemical, and molecular mechanisms. In wheat, some of these responses include inhibition of plant growth and photosynthetic capacity, together with a wide range of physiological responses, including changes in stomatal closure and decreases in transpiration, Rubisco efficiency, and chlorophyll content as well as an increase in oxidative stress among other responses (Budak et al., 2013; Nezhadahmadi et al., 2013). Such responses are modulated by stress severity. Cessation of watering showed a progressive reduction in leaf relative water content, water potential and photosynthesis in durum wheat (Habash et al., 2014). Liu et al. (2016) reported a progressive inhibition of photosynthetic activity as water stress is more severe in field-grown bread wheat, probably due to non-stomatal limitations, which led to lower grain yields even at moderate water stress. Furthermore, water stress in wheat leads to complex changes in the expression of some genes, including those involved in photosynthesis, respiration, N metabolism, lipid metabolism, transcription factors, signal transducers, and synthesis of protective proteins (Habash et al., 2009, 2014; Budak et al., 2013; Yousfi et al., 2016). These changes in gene expression occurred mainly in the early phases of the stress (Habash et al., 2014).

Plant responses to elevated [CO_2_] or water stress are influenced by the duration and level of the environmental factor, the growth stage, and the genetic variability. Studies carried out with different durum wheat genotypes demonstrated that the responsiveness to elevated [CO_2_] (Aranjuelo et al., 2013), water stress (De Leonardis et al., 2007; Aprile et al., 2013; Habash et al., 2014), and the combination of both (Erice et al., 2014) is genotype specific. Moreover, the growth stage greatly influences the response of durum wheat to elevated [CO_2_] (Aranjuelo et al., 2011; Vicente et al., 2015a) and drought (Liu et al., 2016). In addition, the interactive effects of environmental conditions and genotypic variability cannot be anticipated from the individual effects of these treatments (Ceccarelli et al., 1991). Some studies have shown positive effects of elevated [CO_2_] on water stress tolerance of different bread wheat varieties (Harnos et al., 2002; Wall et al., 2006; Robredo et al., 2011; Bencze et al., 2014). A positive synergistic effect of elevated [CO_2_] and water stress has been reported to decrease g_s_, and thus leads to an improvement in water use efficiency at the stomatal and whole plant level (Bencze et al., 2014; Pazzagli et al., 2016). The decrease in photosynthesis under water stress is often mitigated by elevated [CO_2_] (Bencze et al., 2014), resulting in increased levels of carbohydrates for the development of new tissues or filling grain (Wall et al., 2006). However, such positive effects of elevated [CO_2_] in improving stress tolerance are not always achieved (Hudak et al., 1999; Pleijel et al., 2000). Bencze et al. (2014) reported that drought at elevated [CO_2_] led to a stimulation of the antioxidant enzyme system in bread wheat, which suggests a high level of oxidative stress. Erice et al. (2014) showed that the stimulation of plant growth by elevated [CO_2_] was only found in durum wheat genotypes with high harvest indices and optimal water supply. Therefore, additional efforts are still necessary to deepen our understanding of the interactive effect of [CO_2_] and water regime in durum wheat.

The aim of this work was to determine the physiological and molecular mechanisms involved in the adaptive response of four semi-dwarf (i.e., post-Green Revolution) durum wheat cultivars to different [CO_2_] and water regimes. Durum wheat genotypes were grown under controlled conditions at ambient and elevated [CO_2_] and two different water regimes (fully irrigated and moderate/severe water stress). We assessed plant growth, physiological traits, stable C and N isotopic signatures, and transcript levels for stress-responsive genes that could be good indicators of durum wheat's adaptation to future climate conditions at vegetative growth stages. The genes selected corresponded to key enzymes in the metabolism of C (the Rubisco large and small subunits, RBCL and RBCS, respectively, and phosphoenolpyruvate carboxylase, PEPC) and N (the cytosolic and plastidial glutamine synthetases, GS1 and GS2, respectively), as well as proteins involved in stress responses (dehydrins 11, DHN11, and 16, DHN16, catalase, CAT, and superoxide dismutase, SOD). Rubisco is the key enzyme for photosynthetic CO_2_ assimilation, and its activity is highly responsive to atmospheric [CO_2_] (Vicente et al., 2011; Carmo-Silva et al., 2015). PEPC is a cytosolic enzyme that catalyzes the β-carboxylation of phosphoenolpyruvate to produce oxaloacetate, which is involved in anaplerotic functions. GS1 and GS2 play a central role in N metabolism: the former is thought to be involved in the primary assimilation of ammonium from nitrate reduction and photorespiration, while the latter is mainly involved in the transport of N through the plant and N recycling from catabolic processes. The function of the dehydrin family is not completely understood, but these proteins are involved in conferring stress tolerance (Kosová et al., 2014). Catalases and superoxide dismutases are primary antioxidant enzymes involved in the elimination of reactive oxygen species (ROS) such as the cytotoxic H_2_O_2_ produced by photorespiration (Luna et al., 2005) and the superoxide generated during photosynthetic electron transport (Xu et al., 2010; Huseynova et al., 2014). Thus, our study combines the effects of genotypic variability and future environmental conditions, integrating plant performance with gene expression, and aims to identify traits associated with better performance during vegetative growth.

## Materials and methods

### Plant material and growth conditions

The experiment was conducted with four semi-dwarf durum wheat [*Triticum turgidum* L. ssp. *durum* (Desf.)] genotypes: Mexa (year of commercial release: 1977), Regallo (1988), Burgos (1997) and Ramirez (2006). These cultivars represent high-yield genotypes released in the last forty years that are (or were) widely cultivated in the Mediterranean regions of Spain. The study of these genotypes could provide information about the adaptation of modern cultivars to climate change and whether there are differences between them associated with the year they were released. The experiment was conducted from May to July 2015 in two controlled environment chambers (Conviron E15; Controlled Environments, Winnipeg, MB, Canada) in the Experimental Facilities of the Faculty of Biology at the University of Barcelona. A total of 96 durum wheat plants (24 for each genotype) were sown in 2 L pots containing a mixture of standard substrate:perlite (1:1, v/v) and were grown with a long light period of 16 h, a photosynthetic photon flux density (PPFD) of 350 μmol m^−2^ s^−1^, a day/night temperature of 23/17°C and a relative humidity of 60%. During the entire experiment, half of the pots were cultivated under atmospheric [CO_2_] (400 μmol mol^−1^) in one chamber, while the other half grew under elevated [CO_2_] (790 μmol mol^−1^) in the other chamber with injection of CO_2_ from an external bottle (Carburos Metálicos S.A., Barcelona, Spain). The temperature, relative humidity and [CO_2_] within each chamber were continuously monitored by Conviron series controllers (CMP3243 Controlled Environments Ltd., Winnipeg, MB, Canada). The technical staff of the Experimental Facilities of the Faculty of Biology tested the growth conditions of each chamber periodically with external sensors: an HMP75 humidity and temperature probe and a GMP222 CO_2_ probe for use with an MI70 series hand-held indicator (Vaisala, Vantaa, Finland). Similarly, the PPFD was periodically verified with an LI-188B quantum/radiometer/photometer (LI-COR Inc., Lincoln, NB, USA).

**Figure 1:**
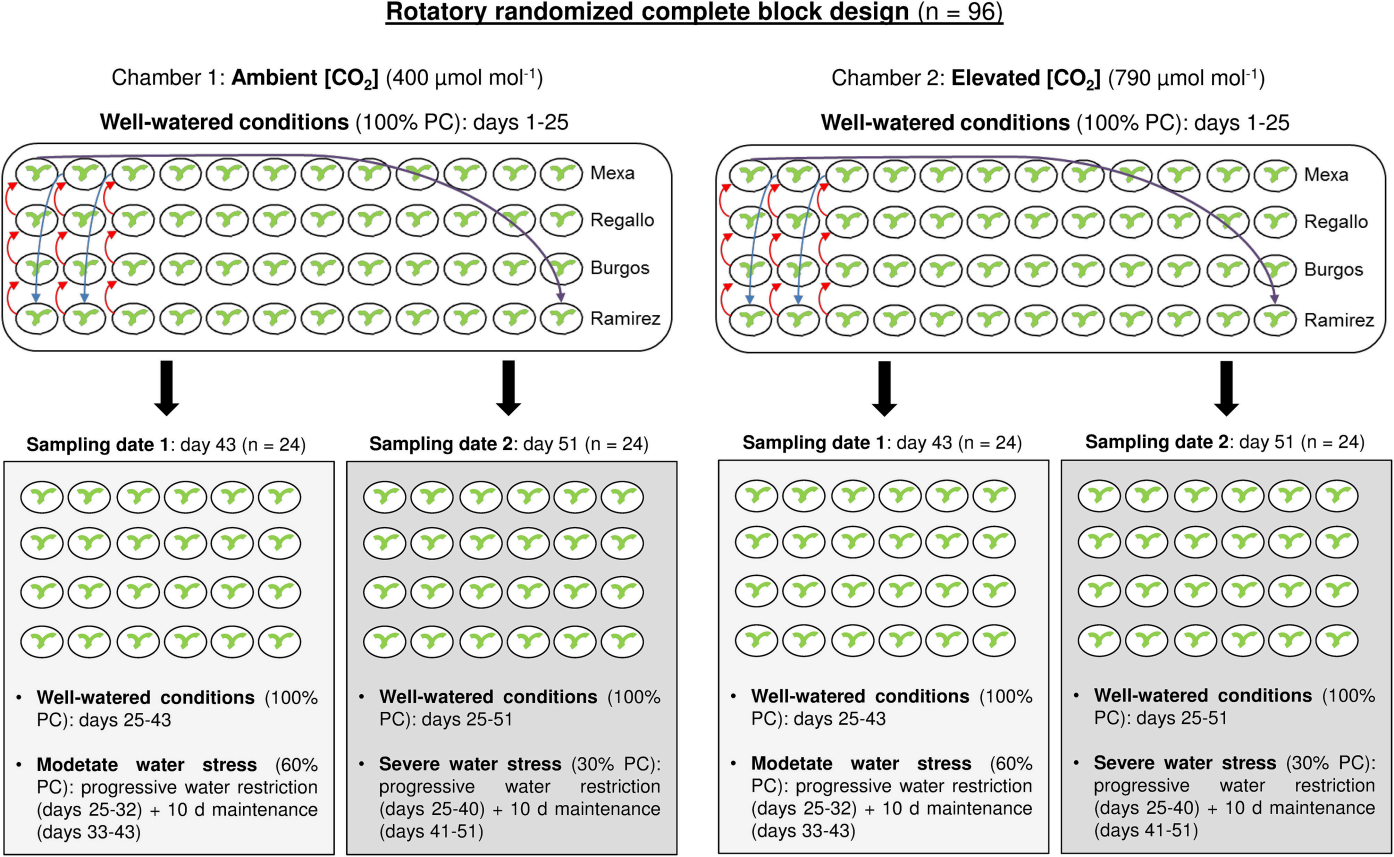
**Schematic representation of the experimental design**.

The plants were uniformly irrigated every 2 days with 50% Hoagland's nutrient solution over a 25 day period. After that (Zadoks 21), the water stress was imposed; one half of the plants of each genotype and [CO_2_] were maintained under well-watered conditions (100% pot capacity, PC) until the end of the experiment, while the other half were subjected to water stress conditions. The maximum soil volumetric water content of each pot was evaluated at the beginning of the experiment as the difference between pot weight after watering with the excess water drained and the pot dry weight. Thus, pots were watered by direct measurements of the pot weight and the water supply was adjusted to the pot water conditions established for each water regime. In the water-stressed plants the watering was progressively restricted by 10% PC every 2 days. First, after 8 days the water-stressed plants received a 60% PC (moderate water stress) and this irrigation regime was strictly maintained for 10 days (see Figure 1 for a schematic representation of the experimental design). At the end of this period (Zadoks 26), equal numbers (48) of well-watered and water-stressed plants were sampled. The youngest fully expanded leaf was collected, immediately frozen in liquid nitrogen and stored at −80°C for gene expression, C and N content and stable isotope analyses. After that, the whole plant was harvested and dried in an oven at 60°C for 72 h for biomass analysis. Second, in the remaining half of the plants (48), the progressive water limitation continued for 8 more days until water-stressed plants received a 30% PC (severe water stress). As in the moderate water stress, the irrigation conditions in well-watered and water-stressed plants were maintained for 10 days. Later, these 51-day-old plants (Zadoks 28–32) were collected following the procedure described above. The moderate and severe water stresses were defined in this experiment based on similar reductions in irrigation and stomatal conductance used in other studies (Galmes et al., 2007; Liu et al., 2016). The pots were rotated three times a week to avoid edge effects in the growth chambers over the course of the experiment. We used a rotatory randomized complete block design with three replicates (one plant per pot) per factor combination ([CO_2_], water level and genotype) at each sampling.

### Physiological traits

Prior to harvest a hand-held portable spectroradiometer (GreenSeeker, NTech Industries, Ukiah, CA, USA) was used to estimate the normalized difference vegetation index (NDVI) of each plant (only at the second sampling date). Relative chlorophyll content was measured with a Minolta SPAD-502 chlorophyll meter (Spectrum Technologies, Plainfield, IL, USA). Stomatal conductance (g_s_) was measured using a Decagon SC-1 Leaf Porometer (Decagon Device, Inc., Pullman, WA, USA). Both chlorophyll content and stomatal conductance of the adaxial surface were recorded in the central segment of the same youngest fully expanded leaf between 3 and 5 h after the start of the photoperiod. In addition, plants were collected to determine the leaf, shoot, root, and plant dry weights as indicated above, while the roots were washed in tap water until all substrate was removed. The number of tillers and the root to shoot dry weight ratio (root/shoot) were then determined.

### C and N content and stable isotope signatures

A fraction of the youngest fully expanded leaf was finely powdered and then 1 mg of this leaf material was used for the measurements of total C and N content (as a percentage of leaf dry weight) and the stable C (^13^C/^12^C) and N (^15^N/^14^N) isotope ratios. Measurements were carried out using an elemental analyzer (Flash 1112 EA; ThermoFinnigan, Bremen, Germany) coupled with an isotope ratio mass spectrometer (Delta C IRMS; ThermoFinnigan), operating in continuous flow mode, at the Scientific Facilities of the University of Barcelona. As has been described previously (Bort et al., 2014; Yousfi et al., 2016), the ^13^C/^12^C ratio was expressed in δ notation: δ^13^C (‰) = [(^13^C/^12^C)_sample_/(^13^C/^12^C)_standard_ − 1] × 1000. The standard refers to international secondary standards of known ^13^C/^12^C ratios (IAEA CH7 polyethylene foil, IAEA CH6 sucrose, and USGS 40 L-glutamic acid) calibrated against Vienna Pee Dee Belemnite calcium carbonate. The same δ notation was used for the ^15^N/^14^N ratio (δ^15^N) using N_2_ in air as standard.

### Quantitative reverse transcriptase PCR amplification

Frozen leaf samples were ground with liquid nitrogen and subsequently RNA was isolated from 100 mg of this material with Ribozol RNA Extraction Reagents (Amresco, Solon, OH, USA) according to the manufacturer's instructions. RNA quantity and quality was measured using a NanoDrop ND-1000 spectrophotometer (Thermo Fisher Scientific, Waltham, MA, USA). RNA integrity was checked by 1.5% (w/v) agarose gel electrophoresis. Total RNA (1 μg) was treated with PerfeCTa DNase I RNase-free (Quanta Biosciences, Gaithersburg, MD, USA) to eliminate residual genomic DNA. cDNA was synthesized using a qScript cDNA Synthesis Kit (Quanta Biosciences) following the manufacturer's instructions. The qRT-PCR assays were performed in optical 384-well-plates with the LightCycler 480 System (Roche Applied Science, Penzberg, Germany) in the Centres Científics i Tecnològics de la Universitat de Barcelona (CCiTUB), in a reaction volume of 10 μL: 5 μL of PerfeCTa SYBR Green FastMix (Quanta Biosciences), 200 nM of each gene-specific primer and 1 μL of diluted cDNA (1:10). The thermal profile was as follows: initial denaturation for 30 s at 95°C, PCR cycling (45 cycles) for 5 s at 95°C, 15 s at 60°C, and 10 s at 72°C, and a final step of 95°C for 5 s and 60°C for 60 s to obtain the dissociation curve. Two technical replicates were analyzed per biological replicate. Specific primers for genes encoding the Rubisco large subunit (NC_021762), phosphoenolpyruvate carboxylase (Y15897), plastidial glutamine synthetase (DQ124212), dehydrin 11 (AJ890140), and superoxide dismutase (KP696754) were designed in Primer-BLAST (http://www.ncbi.nlm.nih.gov/tools/primer-blast/) using the following criteria: Tm = 60 ± 1°C, primer length of 18–25 bases, GC content of 30–70% and product size of 60–150 bases. The specificity of PCR amplification was confirmed by the presence of unique amplicons of the expected length on 3.5% (w/v) agarose gels. The genes encoding the ADP-ribosylation factor and the RNase L inhibitor-like protein, previously identified as potential reference genes (Vicente et al., 2015b), were used to normalize qRT-PCR data after the evaluation of their expression stability in this study. All primers used for gene expression analysis and their symbols are listed in Supplementary Table S1. The values of the cycle threshold (C_*t*_) were calculated using the LightCycler 1.5 software (Roche Applied Science). The quantification of the relative gene expression was analyzed using the comparative C_*t*_ method 2^−ΔΔCt^ (Schmittgen and Livak, 2008), and the data were presented as the log_2_ fold change.

### Data analysis

The effects of [CO_2_] (ambient and elevated), water regime (well-watered and water stressed), genotype (Mexa, Regallo, Burgos, and Ramirez), and their interaction on plant growth, chlorophyll content, g_s_, and C and N contents and isotope composition were determined through a three-factor (2 CO_2_ × 2 water regimes × 4 genotypes) analysis of variance (ANOVA) for each sampling date (moderate and severe waters stress; see Supplementary Table S2) with GenStat 6.2 (VSN International Ltd, Hemel Hempstead, UK). Further, and given the implicit complexity of the design, each genotype was analyzed through a two-factor ANOVA (2 CO_2_ × 2 water regimes) for both sampling dates. All factors were treated as fixed independent variables. When the *F*-ratio was significant (*P* < 0.05), the least significant difference (LSD) test was used to assess differences between treatment means. Clustered heat maps of relative gene expression were built in the R statistics environment (R Development Core Team, 2008) to study the effects of elevated [CO_2_] and water stress on transcript levels. A correlation matrix was generated in R for evaluating the relationships between all parameters analyzed. Visualization of significant correlations was performed using Cytoscape software (Shannon et al., 2003).

## Results

### Effect of [CO_2_], water regime, and genotype on plant growth

Total biomass of the plant and its different fractions (leaves, shoot and root), the root/shoot ratio, and the number of tillers were analyzed through two-factor ANOVA ([CO_2_] × water regime) for each genotype (Tables 1, 2). Moderate and severe water stress were established with reductions of 40 and 70% in the water supplied to the pots and average decreases of 34 and 57% in g_s_, respectively, compared to well-watered plants (data not shown). Growth under elevated [CO_2_] led to significant increases in biomass compared to ambient [CO_2_] (Tables 1, 2). At the first sampling date, elevated [CO_2_] increased root biomass in Mexa, Regallo and Ramirez (and also in Burgos, *P* = 0.074), but only increased plant biomass in Regallo. The root/shoot ratio also increased in Regallo and Burgos under elevated [CO_2_]. At the second sampling date, elevated [CO_2_] increased plant biomass due to higher shoot and root biomass compared to ambient [CO_2_], with larger increases in plant biomass in Mexa and Regallo under well-watered conditions in comparison to water stressed conditions. As a consequence of the increases in both shoot and root dry weights by elevated [CO_2_], the root/shoot ratio was not altered, except in Regallo. Moreover, in this genotype an increase in the tillers per plant was also observed under elevated [CO_2_] but only in well-watered conditions.

**Table 1:** **Total leaf (LDW), shoot (SDW), root (RDW) and plant (PDW) dry weight, root/shoot ratio, and number of tillers per plant in four durum wheat genotypes grown under ambient or elevated [CO_2_] and well-watered or moderate water stress conditions (100 vs. 60% pot capacity)**.

	**Genotype**	**Ambient [CO_2_]**		**Elevated [CO_2_]**		***P*_C_**	***P*_W_**	***P*_C × W_**
		**Well-watered**	**Water stressed**	**Well-watered**	**Water stressed**			
LDW (g)	Mexa	1.84	1.29	1.45	1.23	0.527	0.304	0.656
	Regallo	1.26^a^	1.37^ab^	1.59^b^	1.18^a^	0.402	0.105	**0.013**
	Burgos	1.32	2.36	1.83	1.28	0.562	0.618	0.128
	Ramirez	1.98	1.41	1.70	1.15	0.417	0.109	0.964
SDW (g)	Mexa	3.12	1.85	2.17	2.27	0.732	0.463	0.387
	Regallo	1.75	1.97	2.14	1.90	0.441	0.962	0.258
	Burgos	4.18	3.78	2.47	1.91	0.147	0.679	0.946
	Ramirez	3.27	2.36	2.39	1.64	0.339	0.320	0.924
RDW (g)	Mexa	0.65	0.47	1.24	0.82	**0.023**	0.109	0.512
	Regallo	0.65	0.65	0.96	1.26	<**0.001**	0.080	0.074
	Burgos	0.85	0.49	0.90	1.00	0.074	0.374	0.125
	Ramirez	0.72	0.62	1.10	1.00	**0.022**	0.459	0.981
PDW (g)	Mexa	3.77	2.32	3.41	3.10	0.803	0.298	0.490
	Regallo	2.41	2.62	3.10	3.16	**0.002**	0.351	0.576
	Burgos	5.03	4.27	3.36	2.91	0.211	0.602	0.895
	Ramirez	3.99	2.98	3.50	2.64	0.577	0.232	0.921
Root/shoot	Mexa	0.30	0.26	0.56	0.36	0.055	0.184	0.353
	Regallo	0.38	0.37	0.45	0.69	**0.045**	0.214	0.180
	Burgos	0.28	0.19	0.37	0.52	**0.025**	0.711	0.146
	Ramirez	0.32	0.34	0.47	0.61	0.095	0.484	0.588
Tiller/plant	Mexa	9.3	7.0	8.0	6.7	0.543	0.200	0.713
	Regallo	7.0	8.3	8.3	8.3	0.567	0.567	0.567
	Burgos	8.7	11.0	9.0	9.0	0.620	0.491	0.491
	Ramirez	11.7	8.7	7.0	6.3	**0.045**	0.248	0.451

Significant effects for elevated [CO_2_] (C), water stress (W) and their interaction (C × W) were determined by two-factor ANOVA (P). Values with the same letter are not significantly different for the interaction [CO_2_] × water level. Significant P values are marked in bold (P < 0.05).

**Table 2:** **Total leaf (LDW), shoot (SDW), root (RDW) and plant (PDW) dry weight, root/shoot ratio, number of tillers per plant, and normalized difference vegetation index (NDVI) in four durum wheat genotypes grown under ambient or elevated [CO_2_] and well-watered or severe water stress conditions (100 vs. 30% pot capacity)**.

	**Genotype**	**Ambient [CO_2_]**		**Elevated [CO_2_]**		***P*_C_**	***P*_W_**	***P*_C × W_**
		**Well-watered**	**Water stressed**	**Well-watered**	**Water stressed**			
LDW (g)	Mexa	2.40^a^	2.69^a^	4.73^b^	1.97^a^	0.127	**0.031**	**0.012**
	Regallo	1.09^a^	1.75^b^	4.56^c^	2.23^b^	<**0.001**	**0.001**	<**0.001**
	Burgos	2.77	1.70	4.67	1.80	0.082	**0.004**	0.112
	Ramirez	2.27	1.86	3.28	1.98	0.178	0.057	0.282
SDW (g)	Mexa	4.26^a^	4.96^a^	8.35^b^	4.97^a^	**0.039**	0.147	**0.040**
	Regallo	1.49^a^	3.71^b^	6.24^*c*^	3.20^b^	**0.001**	0.361	<**0.001**
	Burgos	4.78	4.21	8.43	5.43	0.058	0.143	0.301
	Ramirez	3.59	3.53	6.98	3.73	**0.035**	**0.048**	0.054
RDW (g)	Mexa	1.31^a^	1.14^a^	2.65^b^	1.47^a^	**0.002**	**0.008**	**0.030**
	Regallo	2.42	1.92	3.54	2.45	**0.002**	**0.002**	0.139
	Burgos	1.54	1.33	3.01	1.75	**0.003**	**0.012**	0.053
	Ramirez	1.59	1.37	2.33	1.93	**0.013**	0.166	0.689
PDW (g)	Mexa	5.56^a^	6.10^a^	10.99^b^	6.44^a^	**0.018**	0.074	**0.031**
	Regallo	3.91^a^	5.63^b^	9.78^*c*^	5.65^b^	<**0.001**	**0.025**	<**0.001**
	Burgos	6.32	5.54	11.43	7.19	**0.011**	**0.039**	0.128
	Ramirez	5.18	4.89	9.32	5.66	**0.012**	**0.032**	0.058
Root/shoot	Mexa	0.33	0.23	0.32	0.30	0.400	0.123	0.267
	Regallo	1.65^b^	0.58^a^	0.57^a^	0.76^a^	**0.011**	**0.013**	**0.002**
	Burgos	0.40	0.48	0.36	0.34	0.563	0.850	0.718
	Ramirez	0.58	0.39	0.34	0.53	0.662	0.994	0.136
Tiller/plant	Mexa	9.3	9.3	11.0	7.0	0.852	0.279	0.279
	Regallo	6.3^a^	8.0^a^	15.7^b^	10.0^a^	**0.002**	0.138	**0.017**
	Burgos	12.3	7.0	13.0	8.7	0.377	**0.005**	0.699
	Ramirez	8.0	7.7	6.7	6.7	0.773	0.174	0.267
NDVI	Mexa	0.29	0.18	0.36	0.13	0.678	<**0.001**	0.104
	Regallo	0.29	0.18	0.34	0.15	0.610	<**0.001**	0.067
	Burgos	0.25	0.17	0.30	0.16	0.524	**0.006**	0.347
	Ramirez	0.29	0.18	0.25	0.16	0.287	**0.003**	0.651

Significant effects for elevated [CO_2_] (C), water stress (W) and their interaction (C × W) were determined by two-factor ANOVA (P). Values with the same letter are not significantly different for the interaction [CO_2_] × water level. Significant P values are marked in bold (P < 0.05).

Moderate water stress did not lead to statistical differences in biomass, the root/shoot ratio, or the number of tillers between well-watered and water-stressed plants (Table 1). However, severe water stress led to significant changes in these parameters, while NDVI was also affected (Table 2). Plant biomass generally decreased under severe water stress compared to well-watered conditions and was associated with decreases in leaf, shoot, and root dry weights. Water restriction decreased the number of tillers per plant in Burgos under severe water stress, while this reduction was not significant in the other genotypes. Additionally, the NDVI values were lower in water-stressed plants compared to well-watered plants, irrespective of the [CO_2_] and the genotype (Table 2). In general, at severe water stress the interaction [CO_2_] × water regime × genotype showed that the root/shoot ratio strongly increased in Regallo, especially under ambient [CO_2_] and well-watered conditions (Supplementary Table S2). The Burgos and Mexa cultivars had higher shoot dry weight than Ramirez and Regallo, while root dry weight was higher in Regallo (Supplementary Table S2). Furthermore, significant [CO_2_] × genotype interaction showed that Burgos and Regallo under elevated [CO_2_] increased tiller production, whereas Ramirez and Regallo plants under ambient [CO_2_] had lower tillering (Supplementary Table S2).

### Effect of [CO_2_], water regime, and genotype on chlorophyll content, g_s_, C and N content and c and n isotope composition

The interactive effects of [CO_2_] and water regime on chlorophyll content, g_s_, and C and N contents and isotope composition were analyzed in the youngest fully expanded leaf through two-factor ANOVA for each genotype during vegetative growth under moderate (Table 3) and severe water stress (Table 4). At moderate water stress, elevated [CO_2_] compared to ambient [CO_2_] decreased N content in Mexa and Regallo, and δ^13^C regardless of the genotype, while it increased chlorophyll content in Mexa, g_s_, and C content in Regallo, and δ^15^N in all genotypes except in Regallo. Water stress reduced g_s_ and increased δ^15^N in Regallo, and increased δ^13^C in Burgos and Ramirez. Three-factor ANOVA showed significant interactions for δ^15^N (Supplementary Table S2). The [CO_2_] × genotype interaction mainly showed that δ^15^N was higher in Ramirez and Burgos at elevated [CO_2_] and in Regallo at both [CO_2_], whereas the lowest values were observed in Ramirez at ambient [CO_2_]. The water regime × genotype interaction indicated that δ^15^N was higher in Mexa and Regallo under water stress than in the other genotypes.

**Table 3:** **Chlorophyll content, stomatal conductance (g_s_), N and C content, and N and C isotope composition (δ^15^N and δ^13^C, respectively) in four durum wheat genotypes grown under ambient or elevated [CO_2_] and well-watered or moderate water stress conditions (100 vs. 60% pot capacity)**.

	**Genotype**	**Ambient [CO_2_]**		**Elevated [CO_2_]**		***P*_C_**	***P*_W_**	***P*_C × W_**
		**Well-watered**	**Water stressed**	**Well-watered**	**Water stressed**			
Chlorophyll (SPAD units)	Mexa	46.0	51.3	52.3	52.5	**0.049**	0.122	0.156
	Regallo	45.8	52.2	50.5	47.7	0.962	0.472	0.093
	Burgos	45.0	49.4	51.3	50.8	0.178	0.470	0.369
	Ramirez	47.2	41.3	49.3	48.5	0.099	0.216	0.337
g_s_(mmol m^−2^ s^−1^)	Mexa	260.1	58.5	246.9	201.2	0.466	0.183	0.384
	Regallo	248.2	100.9	315.0	262.4	**0.003**	**0.006**	0.114
	Burgos	255.1	105.4	180.3	144.8	0.680	0.056	0.206
	Ramirez	248.6	140.0	166.1	253.1	0.772	0.839	0.093
N (%)	Mexa	5.04	4.79	3.85	4.34	**0.016**	0.675	0.212
	Regallo	5.14	4.72	3.81	3.84	**0.007**	0.534	0.477
	Burgos	5.05	5.16	3.63	4.78	0.174	0.323	0.410
	Ramirez	4.92	4.58	4.52	3.63	0.069	0.092	0.413
δ^15^N(‰)	Mexa	2.43	3.18	3.81	3.78	**0.024**	0.344	0.301
	Regallo	2.35	4.43	3.15	3.34	0.741	**0.029**	0.058
	Burgos	2.19	2.26	3.92	3.18	<**0.001**	0.152	0.087
	Ramirez	2.01	1.98	3.38	3.56	**0.002**	0.831	0.744
C (%)	Mexa	40.6	41.3	41.5	41.0	0.527	0.842	0.158
	Regallo	38.9	39.3	41.0	40.8	**0.015**	0.821	0.577
	Burgos	40.1	40.1	40.3	45.2	0.229	0.268	0.268
	Ramirez	42.1	40.6	40.3	36.8	0.185	0.227	0.620
δ^13^C(‰)	Mexa	−32.8	−29.7	−52.7	−55.5	<**0.001**	0.981	0.483
	Regallo	−33.4	−33.9	−53.9	−60.7	<**0.001**	0.205	0.265
	Burgos	−32.7	−29.0	−57.9	−50.4	<**0.001**	**0.016**	0.337
	Ramirez	−32.5	−29.8	−60.5	−52.8	<**0.001**	**0.047**	0.286

Significant effects for elevated [CO_2_] (C), water stress (W) and their interaction (C × W) were determined by two-factor ANOVA (P). Significant P values are marked in bold (P < 0.05).

**Table 4:** **Chlorophyll content, stomatal conductance (g_s_), N and C content, and N and C isotope composition (δ^15^N and δ^13^C, respectively) in four durum wheat genotypes grown under ambient or elevated [CO_2_] and well-watered or severe water stress conditions (100 vs. 30% pot capacity)**.

	**Genotype**	**Ambient [CO_2_]**		**Elevated [CO_2_]**		***P*_C_**	***P*_W_**	***P*_C × W_**
		**Well-watered**	**Water stressed**	**Well-watered**	**Water stressed**			
Chlorophyll (SPAD units)	Mexa	55.8	55.0	52.7	52.7	0.100	0.790	0.807
	Regallo	50.6	43.1	46.6	45.8	0.877	0.351	0.439
	Burgos	56.6	53.2	53.3	58.4	0.661	0.683	0.076
	Ramirez	52.7	51.6	52.8	49.0	0.532	0.230	0.500
g_s_(mmol m^−2^ s^−1^)	Mexa	81.9	55.0	223.5	91.5	0.112	0.150	0.323
	Regallo	101.8	82.3	64.4	32.2	0.265	0.499	0.866
	Burgos	245.0	96.7	247.0	30.7	0.390	<**0.001**	0.362
	Ramirez	44.0	52.5	151.3	57.2	0.160	0.270	0.194
N (%)	Mexa	4.39	4.67	4.10	4.54	0.491	0.257	0.785
	Regallo	4.68	4.33	4.37	3.78	**0.017**	**0.011**	0.424
	Burgos	4.89	4.66	4.15	3.97	**0.013**	0.398	0.918
	Ramirez	3.81	4.22	4.16	4.43	0.521	0.437	0.872
δ^15^N(‰)	Mexa	2.71	2.74	3.52	3.59	0.060	0.895	0.950
	Regallo	3.78	3.27	3.55	3.58	0.947	0.685	0.646
	Burgos	2.45	2.67	3.69	3.47	**0.018**	0.997	0.547
	Ramirez	2.69	2.99	3.23	2.73	0.508	0.641	0.084
C (%)	Mexa	41.8	41.8	41.3	41.9	0.490	0.368	0.439
	Regallo	39.9	38.0	40.4	41.1	0.064	0.476	0.165
	Burgos	41.9	41.4	41.8	41.3	0.877	0.590	0.968
	Ramirez	41.4	40.7	40.9	42.1	0.416	0.658	0.115
δ^13^C(‰)	Mexa	−32.3	−31.4	−56.9	−56.4	<**0.001**	0.546	0.851
	Regallo	−33.5	−32.2	−54.4	−54.0	**0.001**	0.850	0.927
	Burgos	−32.8	−32.4	−46.1	−46.3	**0.004**	0.979	0.940
	Ramirez	−31.9	−32.6	−59.1	−56.4	<**0.001**	0.752	0.594

Significant effects for elevated [CO_2_] (C), water stress (W) and their interaction (C × W) were determined by two-factor ANOVA (P). Significant P values are marked in bold (P < 0.05).

At severe water stress, elevated [CO_2_] relative to ambient [CO_2_] decreased the N content in Regallo and Burgos and δ^13^C regardless of the genotype, while it increased δ^15^N in Burgos (Table 4). Furthermore, g_s_ in Burgos and N content in Regallo decreased under severe water stress compared to well-watered conditions. In addition, under well-watered conditions g_s_ was higher in Burgos than in other genotypes (Supplementary Table S2). Chlorophyll content was lower in Regallo, whereas δ^13^C was higher in Burgos, as compared to other genotypes (Supplementary Table S2).

### Effect of [CO_2_] and water regime on gene expression for each durum wheat genotype

Treatment effects on transcript levels were evaluated for each genotype using nine genes that encode enzymes of primary C and N metabolism and stress-responsive proteins (Supplementary Table S1). Elevated [CO_2_] and water stress led to changes in gene expression depending on genotype and the level of water restriction (Table 5; Supplementary Figure S1). At moderate water stress, elevated [CO_2_] decreased transcript levels of RBCL, RBCS, and GS2 relative to control conditions (ambient [CO_2_] and well-watered conditions), particularly in the Mexa and Regallo genotypes. Under water stress the transcript levels for these enzymes markedly increased in Ramirez. Elevated [CO_2_] caused a generalized increase in the transcript levels of PEPC and GS1, particularly when it was combined with moderate water stress. Transcript abundances of the dehydrins, DHN11 and DHN16, were generally higher under elevated [CO_2_] and well-watered conditions, but lower under ambient [CO_2_] and water stress relative to control conditions. However, *DHN11* and *DHN16* showed opposite expression patterns under elevated [CO_2_] and water stress. [CO_2_] enrichment and moderate water stress decreased transcript levels of CAT and SOD in Mexa, Regallo, and Burgos compared with control conditions, whereas in Ramirez they did not change significantly.

**Table 5:** **Transcript changes in four durum wheat genotypes grown under ambient or elevated [CO_2_] and well-watered or water stressed conditions: (A) moderate and (B) severe water stress**.

**Genotype**	**[CO_2_]**	**Water supply**	**RBCL**	**RBCS**	**PEPC**	**GS1**	**GS2**	**DHN11**	**DHN16**	**CAT**	**SOD**
**(A) MODERATE WATER STRESS (100 vs. 60% POT CAPACITY)**											
Mexa	Ambient [CO_2_]	Water stressed	0.15	0.26	−1.01	−0.88	−0.06	−1.02	−2.83	−2.02	−0.33
Mexa	Elevated [CO_2_]	Well-watered	−0.96	−4.81	0.76	1.93	−3.43	−0.98	1.79	−1.01	−1.06
Mexa	Elevated [CO_2_]	Water stressed	−1.69	−1.66	−2.25	1.65	−1.81	−1.28	−1.02	−2.42	−1.05
Regallo	Ambient [CO_2_]	Water stressed	−0.25	−0.62	0.2	−0.53	−0.27	−0.74	−0.66	−2.42	−0.85
Regallo	Elevated [CO_2_]	Well-watered	−2.59	−1.71	−1.14	−0.35	−1.26	1.26	1.8	−2.31	−0.42
Regallo	Elevated [CO_2_]	Water stressed	−0.59	−0.55	1.52	0.68	−1.29	−0.95	4.81	−1.62	−1.91
Burgos	Ambient [CO_2_]	Water stressed	0.06	0.07	0.19	−0.21	0.93	−0.65	3.27	−2.65	−1.99
Burgos	Elevated [CO_2_]	Well-watered	−2.45	−1.35	0.1	−0.92	−0.87	0.89	1.8	−3.18	−0.35
Burgos	Elevated [CO_2_]	Water stressed	0.47	0.08	2.14	0.67	−0.37	−0.83	2.48	−2.75	−1.76
Ramirez	Ambient [CO_2_]	Water stressed	1.7	1.76	0.63	0.34	1.24	−0.03	−2.1	−0.51	0.49
Ramirez	Elevated [CO_2_]	Well-watered	−0.42	−0.87	0.67	1.03	−0.7	0.38	0.62	−0.47	−0.68
Ramirez	Elevated [CO_2_]	Water stressed	0.56	−1.73	1.99	2.43	−1.21	−1.12	2.49	0.18	0.39
**(B) SEVERE WATER STRESS (100 vs. 30% POT CAPACITY)**											
Mexa	Ambient [CO_2_]	Water stressed	0.57	0.52	0.31	0.19	0.08	−0.37	0.40	0.60	0.25
Mexa	Elevated [CO_2_]	Well-watered	1.84	1.49	0.84	1.09	1.27	−0.23	−1.22	1.56	−0.03
Mexa	Elevated [CO_2_]	Water stressed	−0.34	0.16	−0.42	−0.29	0.13	−0.58	−0.34	1.30	0.04
Regallo	Ambient [CO_2_]	Water stressed	−1.35	−0.59	0.03	−0.19	−0.84	−1.14	1.02	−0.26	0.95
Regallo	Elevated [CO_2_]	Well-watered	−1.38	−0.90	−2.38	−0.61	−0.77	−1.35	−1.69	−0.40	−0.17
Regallo	Elevated [CO_2_]	Water stressed	−0.30	−0.05	−0.59	2.15	−0.44	0.17	3.31	1.80	0.57
Burgos	Ambient [CO_2_]	Water stressed	−0.93	−0.83	−1.98	−3.21	−0.81	−1.35	−6.30	−2.70	0.71
Burgos	Elevated [CO_2_]	Well-watered	−2.13	−1.70	−2.18	−2.75	−1.38	0.19	−7.46	−1.85	−0.02
Burgos	Elevated [CO_2_]	Water stressed	−1.81	−1.73	−2.08	−2.92	−1.05	−0.46	−5.56	−1.91	0.80
Ramirez	Ambient [CO_2_]	Water stressed	−0.44	−0.45	2.34	0.29	0.15	−0.57	1.68	−0.62	0.32
Ramirez	Elevated [CO_2_]	Well-watered	−2.71	−2.34	0.55	1.11	−1.00	−0.04	0.04	−0.18	−1.18
Ramirez	Elevated [CO_2_]	Water stressed	0.51	0.64	0.98	0.25	0.98	1.01	−0.47	0.81	−0.40

White indicates no change, blue up-regulation, and red down-regulation in each treatment relative to the treatment under ambient [CO_2_] and optimal water supply for each genotype, as shown in the color bar for a log_2_ scale. RBCL, Rubisco large subunit; RBCS, Rubisco small subunit; PEPC, phosphoenolpyruvate carboxylase; GS1, cytosolic glutamine synthetase; GS2, plastidial glutamine synthetase; DHN11, dehydrin 11; DHN16, dehydrin 16; CAT, catalase; SOD, superoxide dismutase.

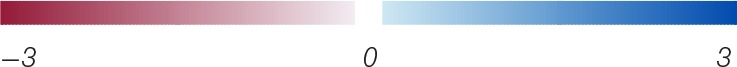

Gene expression analysis indicated greater genotype-specific differences under severe water stress than under moderate water stress (Table 5; Supplementary Figure S1). In Mexa under elevated [CO_2_] and well-watered conditions there were higher transcript levels of RBCL, RBCS, PEPC, GS1, GS2, and CAT and lower levels of DHN16, relative to control conditions. Severe water stress did not substantially alter gene expression. In Regallo most of the transcripts studied were lower in all treatment combinations than in control conditions. However, DHN16 and SOD transcripts increased under ambient [CO_2_] and water stress, and these together with CAT and GS1 also increased under elevated [CO_2_] and water stress. In the case of Burgos, elevated [CO_2_], water stress and their combination strongly reduced transcript levels in comparison to control conditions, especially for GS1 and DHN16, while SOD transcripts increased under water stress and elevated [CO_2_] × water stress as observed in Regallo. In Ramirez elevated [CO_2_] led to a reduction in the transcript levels of RBCL, RBCS, and SOD and an increase in PEPC and GS1 compared to control conditions. Water stress increased PEPC and DHN16 transcript levels relative to control conditions, while under the combination of elevated [CO_2_] and water stress greater transcript abundances were observed for most of the genes.

### Correlation network of physiological traits and gene expression

**Figure 2:**
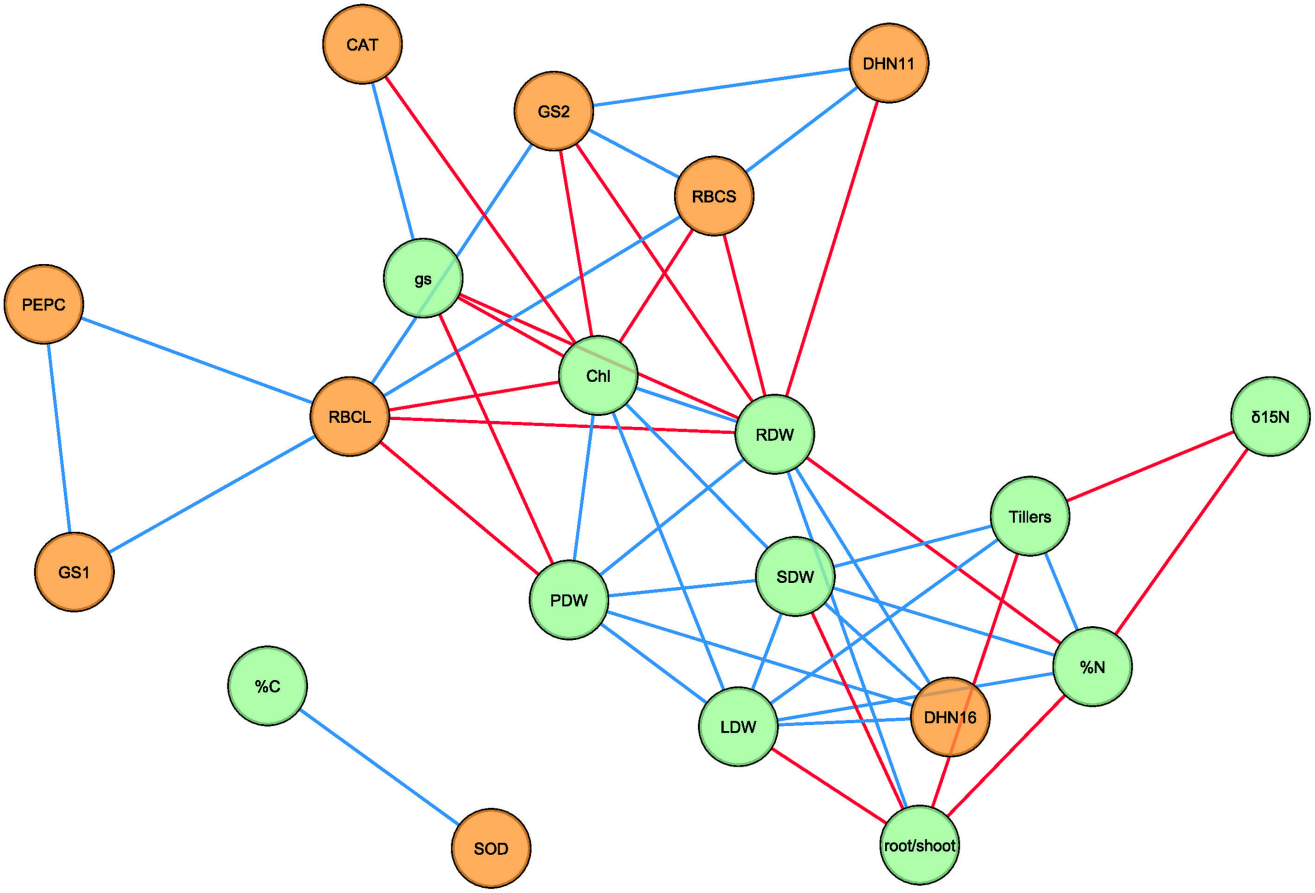
**Network analysis of physiological traits and transcript levels under different [CO_2_] levels, water regimes and sampling dates in four durum wheat genotypes**. The network consists of 20 nodes and 47 edges. Green and orange nodes represent physiological traits and transcript levels, respectively. Blue and red edges represent positive and negative correlations, respectively, based on Pearson's correlation coefficients. For transcript description see the legend of Table 5. Chl, chlorophyll; g_s_, stomatal conductance; LDW, leaf dry weight; PDW, plant dry weight; SDW, shoot dry weight; RDW, root dry weight; %C, carbon content; %N, nitrogen content.

A Pearson correlation matrix was generated using the mean values for each treatment combination, genotype and sampling date (*n* = 32) of the physiological traits and transcript levels (Supplementary Table S3), excluding NDVI, which was only measured at severe water stress, and δ^13^C, which was influenced by C composition of the CO_2_ bottles used in the elevated [CO_2_] chamber (Aljazairi et al., 2015). Of the 190 correlations between parameters, there were 28 positive and 19 negative significant correlations (*P* < 0.05) that are represented in an association network (Figure 2). Most of the significant correlations were observed between physiological traits and transcript levels independently. Positive correlations were found among leaf, shoot, and plant dry weights, between the leaf and shoot dry weights with the number of tillers, and between root and plant dry weights. The root/shoot ratio was positively correlated with root dry weight and negatively correlated with leaf and shoot dry weights, the number of tillers and N content. Furthermore, δ^15^N was also negatively correlated with N content and the number of tillers. Chlorophyll content was correlated positively with leaf, shoot, root, and plant dry weights, and negatively with g_s_. On the other hand, positive correlations were found between N content with leaf and shoot dry weights and the number of tillers, and negative correlations between N content with root dry weight, and between g_s_ with root and plant dry weights. In the case of transcript levels, RBCL was correlated with RBCS, GS1, GS2, and PEPC, whereas RBCS correlated with GS2 and DHN11, GS2 with DHN11, and PEPC with GS1. Furthermore, some relationships were found between physiological traits and gene expression (Figure 2). Positive correlations appeared between DHN16 with plant biomass (leaf, shoot, root, and plant dry weights), CAT with g_s_ and SOD with C content. Moreover, negative correlations were found between chlorophyll content with RBCL, RBCS, GS2, and CAT, also between root dry weight with RBCL, RBCS, GS2, and DHN11, and finally plant dry weight with RBCL.

## Discussion

Although, substantial efforts have been made in recent years to identify traits associated with wheat performance during early growth (Maydup et al., 2012; Rebolledo et al., 2013; Bort et al., 2014; Pang et al., 2014; Wilson et al., 2015), little attention has been paid to the effect of interactions between elevated [CO_2_] and water stress in durum wheat. The effects of water restriction on crop growth have been mostly studied with the view of improving drought impacts at late growth stages in Mediterranean environments. However, projections of future climate change in the Iberian Peninsula predict major rainfall limitations and higher evapotranspiration during winter months (Russo et al., 2015) and therefore early-season drought is a matter of concern. In this context, we describe the effects of elevated [CO_2_] and water stress during the first part of the growth cycle in four durum wheat genotypes on physiological traits and expression of nine genes that respond to changes in [CO_2_] and water levels (Ali-Benali et al., 2005; Budak et al., 2013; Vicente et al., 2015b; Yousfi et al., 2016). The coordination of these parameters under the different combinations of factors is discussed.

### Changes in physiological traits of durum wheat genotypes under different water regimes and [CO_2_] levels

A moderate water stress in 43-day-old plants did not significantly alter plant growth (Table 1). Long-term exposure to elevated [CO_2_] led to higher root biomass relative to ambient [CO_2_] independently of genotypic variability, in concordance with reports from other crop species (Madhu and Hatfield, 2013). This increment was associated with higher plant growth in Regallo and higher root/shoot ratios in Regallo, Burgos, and Mexa (Table 1). In fact, under elevated [CO_2_] root growth is often more stimulated than the aerial part of the plant, although it depends on genotype × environment variation (Stitt and Krapp, 1999; Madhu and Hatfield, 2013). A severe water stress in 51-day-old plants showed greater effects on plant growth than moderate water stress (Table 2). [CO_2_] enrichment generally led to an increase in plant biomass by increasing root and shoot biomass and tillering, particularly under optimal water supply. This could be due to the effects of [CO_2_] fertilization on the net photosynthetic rate (Long et al., 2006; Vicente et al., 2015b), especially in genotypes with large harvest indices such as post-Green Revolution cultivars (Aranjuelo et al., 2013). It could also be caused by carbohydrate accumulation, which may lead to increases in the number of tillers (Stitt and Krapp, 1999). On the other hand, severe water stress constrained plant growth (dry matter and NDVI), in agreement with earlier studies in durum wheat (Erice et al., 2014; Nakhforoosh et al., 2015; Yousfi et al., 2016), with Ramirez and Burgos being the genotypes most affected. According to Marti et al. (2007), we suggest that progressive water restriction during the vegetative stage constrained the photosynthetic area, which may cause negative effects on final biomass and yield.

Chlorophyll content, g_s_, and N and C contents and isotope compositions at moderate and severe water stress did not reveal statistical significance for the interactions [CO_2_] × water regime and [CO_2_] × water regime × genotype (Tables 3, 4; Supplementary Table S2). Stomatal conductance (g_s_) generally decreases under elevated [CO_2_] and drought stress due to an increase in internal [CO_2_] and as a water saving strategy, respectively (Long et al., 2006; Nakhforoosh et al., 2015; Vicente et al., 2015b; Pazzagli et al., 2016). The average g_s_ values decreased under water restriction at moderate and severe water stress, but it was only significantly decreased in some genotypes (Tables 3, 4). On the other hand, elevated [CO_2_] did not alter g_s_ at this growth stage, except for an increase in g_s_ under moderate water stress in Regallo, which could favor CO_2_ assimilation and consequently biomass accumulation under this water regime (Tables 1, 3). Earlier studies have shown a decrease in g_s_ under water stress (Peremarti et al., 2014; Pazzagli et al., 2016), while negligible changes have been reported under elevated [CO_2_] in tomato and durum wheat, and increases have even been recorded for Regallo (Vicente et al., 2015a; Pazzagli et al., 2016). Therefore, the growth stage and the severity of the water stress influenced stomatal closure, while elevated [CO_2_] had minor effects on g_s_ during vegetative growth.

Elevated [CO_2_] generally decreased N content in the present study (Tables 3, 4), which has been observed in C_3_ plants through shifts in N uptake and/or assimilation (which agrees with the changes in transcript levels of N-metabolism enzymes; see below) together with other uncertain mechanisms, e.g., the biomass dilution effect, increased N loss, and sink limitation (Stitt and Krapp, 1999; Aranjuelo et al., 2011; Vicente et al., 2015a,b). N content was also diminished by severe water stress in Regallo, in agreement with previous studies in durum wheat (Yousfi et al., 2012, 2016). Chlorophyll content only increased under elevated [CO_2_] in Mexa at the first sampling date, but the effect disappeared at the second sampling (Table 3). [CO_2_] enrichment and water stress did not modify C content in leaves, suggesting that the decrease in N content was not simply due to N dilution caused by rapid growth (Taub and Wang, 2008). Overall, our data showed that the decrease in N content in plants grown under elevated [CO_2_] and water stress during vegetative growth is genotypically dependent.

The δ^13^C and δ^15^N have been used as potential physiological tracers in plants under elevated [CO_2_] and water limitation (Aranjuelo et al., 2011; Yousfi et al., 2012, 2016; Araus et al., 2013; Bort et al., 2014). Elevated [CO_2_] and water stress caused an increase in δ^15^N, although these effects depended on the genotype and were attenuated or disappeared in severe water stress relative to the moderate stress treatment (Tables 3, 4; Supplementary Table S2). Variations in δ^15^N in response to the growth conditions, together with N content, could indicate shifts in N metabolism (Bort et al., 2014), although δ^15^N is determined by many processes that are not completely understood (Ariz et al., 2015). Nevertheless, the higher δ^15^N could suggest lower N availability, because N absorption and assimilation cannot fractionate between the ^14^N and ^15^N isotopologues under such environmental factors (Lopes and Araus, 2006; Tcherkez, 2011). Additionally, this could reflect a decrease in N translocation from the root to the shoot (Lopes and Araus, 2006). Moreover, δ^13^C increased in some genotypes under moderate water stress, regardless of the [CO_2_] considered, but this increment, also observed under severe water stress, did not reach statistical significance (Tables 3, 4). Elazab et al. (2012) and Bort et al. (2014) also showed a δ^13^C increase in flag leaves of different durum wheat genotypes under water stress at later growth stages, which could be associated with higher water-use efficiency (Araus et al., 2008, 2013; Tardieu, 2013; Bort et al., 2014). A stronger water stress does not always lead to larger changes in δ^13^C, particularly when analyzed in dry matter, as noted in previous studies in rice (Kano-Nakata et al., 2014) and *Pinus tabuliformis* (Ma et al., 2014). In addition, δ^13^C was strongly reduced at high [CO_2_] because of the very negative δ^13^C of the CO_2_ used to increase the [CO_2_] within the growth chamber (Aljazairi et al., 2015).

### Expression of stress-responsive genes in durum wheat genotypes under different water regimes and [CO_2_] levels

Strong differences in gene expression were observed between treatments and among the different genotypes studied (Table 5; Supplementary Figure S1). In our study, RBCL and RBCS showed a common expression pattern (Table 5), confirming the coordinated expression of both subunits necessary for the assembly of the Rubisco holoenzyme (Suzuki and Makino, 2012). At the first sampling date, gene expression of *RBCL* and *RBCS* was down-regulated in response to elevated [CO_2_] no matter which water regime was considered, in agreement with other wheat studies (Aranjuelo et al., 2013; Habash et al., 2014; Vicente et al., 2015b). This down-regulation was associated with lower N content and higher δ^15^N in a genotype-dependent manner. The former could be explained by non-selective decreases in N or reallocation of N within the plant under elevated [CO_2_] (Aranjuelo et al., 2011; Vicente et al., 2015a). The latter was probably associated with changes in N uptake, assimilation or redistribution within the plant (Araus et al., 2013). At the second sampling date, elevated [CO_2_] decreased the N content in Regallo and Burgos, which was related to down-regulation of transcript levels of Rubisco subunits and N-assimilation enzymes (GS1 and GS2), and higher root and plant biomass. These shifts could indicate that plant biomass might increase under elevated [CO_2_] in a genotype-dependent manner even when transcript levels of Rubisco subunits decrease during vegetative growth. This could be due to the remobilization of an N over-investment in Rubisco to reuse it in developing new tissues (Richards, 2000; Vicente et al., 2011; Carmo-Silva et al., 2015). However, the decrease in Rubisco transcript levels under water stress did not indicate the greater photosynthetic efficiency that was hypothesized under elevated [CO_2_]. Instead it was associated with lower plant biomass, which might suggest an inhibition of CO_2_ assimilation and plant growth in concordance with previous studies (Hayano-Kanashiro et al., 2009; Peremarti et al., 2014).

PEPC is a multifaceted key enzyme that in C_3_ plants is linked to the provision of Krebs cycle intermediates, and its overexpression in transgenic wheat improved drought tolerance and grain yield (Qin et al., 2015). *PEPC* expression has not been widely studied during early growth in durum wheat plants. In the current work it was induced under the combination of elevated [CO_2_] and moderate water stress in most genotypes, whereas at severe water stress genotypic variation determined its expression pattern (Table 5). The induction could be related to its major role in providing C skeletons for amino acid and lipid biosynthesis (González et al., 2003). This may be due to an increase in the enzyme's substrates, such as carbohydrates, typically found under elevated [CO_2_] and water stress (Khoshro et al., 2013; Vicente et al., 2015b). These results indicate that further work is necessary to broaden our understanding of the biological role of PEPC and its implication in plant growth, especially in genotypes (i.e., Ramirez) with an up-regulation of gene expression under stress conditions.

At moderate and severe water stress, *GS1* and *PEPC* expression was significantly coordinated, as were the expressions of the *GS2* and Rubisco genes (Table 5; Supplementary Table S3). Under severe water stress, *GS1* and *GS2* expression was more influenced by genotypic variability than environmental conditions. Yousfi et al. (2016) also reported genotypic differences in the expression of these genes under drought stress, with a general down-regulation under stress conditions. Lower N contents and transcript abundances for RBCL and RBCS under water stress and especially under elevated [CO_2_] were associated with higher repression of the *GS2* gene, indicating a coregulation of primary C and N metabolism (Stitt and Krapp, 1999; Vicente et al., 2015b, 2016). In some treatments, mainly at the first sampling date, opposing gene expression patterns were observed between *GS1* and *GS2*. This fact, together with the coordination of *GS1* with *PEPC*, might indicate a predominant remobilization of C and N compounds and an inhibition of primary N assimilation under water stress and elevated [CO_2_]. Thus, the results support a significant coordination between C and N metabolism at the transcript level under conditions of elevated [CO_2_] and water stress. In addition, the pattern of gene expression for *GS1* and *GS2* supports the use of these genes as indicators of N metabolism under water stress conditions, as reported previously (Nagy et al., 2013).

DHN11 and DHN16 encode for two dehydrins that belong to group 2 of late embryogenesis abundant (LEA) proteins (Ali-Benali et al., 2005). The up-regulation of dehydrin genes under water restriction is often associated with stress tolerance, although their specific role as osmotically active compounds is still unknown (Kosová et al., 2014). Moderate and severe water stress reduced *DHN11* gene expression regardless of the [CO_2_] level compared with control conditions. In the case of the *DHN16* gene, moderate water stress mostly up-regulated its expression, whereas under severe water stress the opposite occurred (Table 5). Elevated [CO_2_] at the first sampling date mostly enhanced *DHN11* and *DHN16* gene expression, while at the second sampling date its combination with severe water stress led to a wide range of changes in transcript levels in a genotype-dependent manner. Our results showed that the pattern of gene expression could differ between dehydrins, in concordance with previous studies (Ali-Benali et al., 2005; Melloul et al., 2013; Kosová et al., 2014). Additionally, the severity of the water stress, [CO_2_] enrichment and the genotype influenced dehydrin transcript levels.

CAT and SOD enzymes form part of the system responsible for lowering ROS and avoiding oxidative stress. In general, gene expression of *CAT* and *SOD* was repressed under moderate water stress regardless of [CO_2_] (Table 5). Such repression was only maintained for *CAT* at severe water stress in Burgos, while their expression was up-regulated in the other genotypes under elevated [CO_2_] × severe water stress. This could suggest a higher demand for ROS control, which would indicate a limitation to the transfer of electrons through photosystems to drive C assimilation (Martins et al., 2016). Enzyme activity and *CAT* gene expression have been reported to decrease under elevated [CO_2_] in wheat, possibly due to the inhibition of photorespiration, while they increased only in response to severe drought (Luna et al., 2005; Xu et al., 2010; Vicente et al., 2015b). The available studies reporting changes in *SOD* gene expression and protein content under such conditions are contradictory, reporting different pattern of changes (Kim et al., 2006; Li et al., 2008; Caruso et al., 2009; Xu et al., 2010). Our results highlighted that water regime and genotype were key factors influencing the expression of genes involved in the antioxidant system, indicating a greater need for protection against oxidative damage under severe water stress.

### Coordination between physiological traits and transcript levels in durum wheat grown under different environmental conditions during vegetative growth

The different changes in plant growth parameters indicate that the responsiveness to elevated [CO_2_] and water stress during early growth depends on (i) the duration of the treatment, because [CO_2_] enrichment results in greater increases in plant biomass in older plants; (ii) the severity of the water stress, which is more pronounced under severe water stress; (iii) and the genotypic variability. In general, elevated [CO_2_] stimulated plant growth and reduced N content, which at the transcript level was related to a down-regulation of Rubisco and N assimilation genes and up-regulation of genes that take part in C-N remobilization. Moderate water stress did not lead to gross changes in physiological traits, but severe water stress restricted plant growth and N content, while changes in g_s_ and δ^13^C suggested a water-saving strategy relative to well-watered conditions. The transcript profile suggested an inhibition of primary C fixation and N assimilation, differences between dehydrins and a genotypic variation in gene expression under severe water stress, with an induction of genes involved in antioxidant machinery. The stimulation of plant biomass under elevated [CO_2_] did not compensate for plant growth limitation under water restriction. Lastly, we observed different genotypic responses to environmental factors, as also reported in barley (Ceccarelli et al., 1991). Regallo showed the lowest plant biomass and chlorophyll and N contents, which was related to a repression of genes for N assimilation and induction for dehydrins, SOD and CAT, while the opposite results were recorded for Burgos (data not shown). Therefore, increased plant growth was linked to up-regulation of N assimilation and down-regulation of stress-responsive genes, suggesting lower oxidative damage.

Considering different environmental conditions predicted for the future climate scenario and genotypic variations, network analysis was used to identify physiological traits, and transcript levels that are correlated during vegetative growth in durum wheat (Figure 2). Early growth is a positive trait for improving plant tolerance in water-limited environments that has the potential for larger final plant biomass and yield (Wilson et al., 2015). Plant growth parameters were positively correlated with each other in most cases, suggesting that early plant growth is driven by all plant fractions and tiller production, as reported in other studies (Rebolledo et al., 2013; Wilson et al., 2015). Regardless of genotype, the positive correlation between root and plant biomass was mainly due to the stimulation of root biomass under elevated [CO_2_], in agreement with previous reports (Madhu and Hatfield, 2013; and citations therein). In contrast, water restriction (mainly severe water stress) limited both root and shoot biomass, which are often diminished under severe drought conditions (Nezhadahmadi et al., 2013). Positive effects of elevated [CO_2_] on root biomass could mitigate drought effects on plant growth by allowing better exploitation of water and nutrients from deep soil layers (Madhu and Hatfield, 2013).

N content was correlated negatively with the root/shoot ratio and positively with the tillers per plant and shoot biomass, and this was probably due to the typically higher N content observed in shoots relative to roots (Vicente et al., 2015a). Hence, greater vegetative growth in durum wheat requires high amounts of N, which in turn will be conditioned by N availability. δ^15^N has been proposed as an indicator of responses to stress, such as water stress, N starvation and salinity (Yousfi et al., 2012, 2016; Bort et al., 2014), although it has had little attention for studies of elevated [CO_2_] (Ariz et al., 2015). Here we observed a negative correlation of δ^15^N with N content and tillers per plant, with elevated [CO_2_] being the main factor that increased δ^15^N in our experiment. Nevertheless, the fractionating processes of N metabolism affecting δ^15^N under elevated [CO_2_] and water stress are not fully understood (Tcherkez, 2011).

Leaf chlorophyll content has been extensively used as an indicator of different physiological and agronomical components, particularly at later growth stages (Araus et al., 2008). The network analysis confirmed that chlorophyll content is a positive trait for vegetative growth in durum wheat, and this can be easily implemented in most of studies because this measurement is simple, quick, and non-destructive with modern portable devices. Effects of elevated [CO_2_] and water stress on g_s_ have been widely studied (Long et al., 2006; Pazzagli et al., 2016), including the proposal of g_s_ as a trait indicator of drought stress tolerance (Nagy et al., 2013). In our study g_s_ was negatively correlated with chlorophyll content and root and plant biomass. This could highlight that increased vegetative growth was related to stomatal closure, maybe as a water saving strategy or as a direct response to elevated [CO_2_].

The positive correlations among the transcript levels of the genes encoding RBCL, RBCS, GS1, GS2, and PEPC supported a balanced coordination between C and N metabolism under elevated [CO_2_] and water stress. On the other hand, our results underlined the key role of Rubisco and GS in plant responses to environmental conditions (Nagy et al., 2013; Carmo-Silva et al., 2015; Vicente et al., 2015b; Yousfi et al., 2016). We showed negative associations between transcript levels of Rubisco subunits and GS2 with chlorophyll content and plant biomass. This fact could indicate that a stimulation of plant growth may be associated with a lower investment of resources (mainly N) in Rubisco protein, especially under elevated [CO_2_], thus leading to a higher nitrogen efficiency (Pang et al., 2014; Carmo-Silva et al., 2015). The negative correlation between transcript levels of CAT and chlorophyll content highlighted that the up-regulation of *CAT* expression was a response to the high H_2_O_2_ levels generated under stress conditions (Luna et al., 2005), which could promote chlorophyll degradation (Upadhyaya et al., 2007). Interestingly, transcript levels of CAT were positively correlated with g_s_, although a negative correlation should be expected since greater g_s_ leads to lower photorespiration rates and consequently lower H_2_O_2_ generation (Luna et al., 2005). We found a positive relationship between C content and transcript accumulation for SOD, not previously reported to our knowledge. Higher *SOD* expression might suggest a better ROS control that triggers an efficient electron transfer and C fixation. In our study, DHN11 transcript accumulation was negatively associated with root biomass, while transcripts for DHN16 were positively linked with plant biomass. These results suggest promising functions for DHN16 in stress tolerance during vegetative growth, as Kosová et al. (2014) proposed in a study examining wheat seed development.

In summary, parameters such as chlorophyll and N content, g_s_ and δ^15^N, and the expression of *RBCL, RBCS, GS2, DHN11*, and *DHN16* genes were identified as good indicators for the selection of genotypes with better performance during early plant growth under elevated CO_2_ and water stress. Additionally, network analysis underlined the relevance of N metabolism-traits such as N content, δ^15^N, GS1, and GS2, in the genotypic response of durum wheat to future environmental scenarios in the Mediterranean basin.

## Conclusion

We conclude that [CO_2_] effects on plant growth had greater impacts than moderate or severe water stress during vegetative growth of durum wheat. Whereas, elevated [CO_2_] generally led to increases in plant growth, water stress had a negative effect, preferentially as the water stress develops over time. In addition, the interactive effects of both [CO_2_] and water regime depends on genotypic variability. Gene expression profiles at moderate water stress were mainly affected by environmental conditions among the different genotypes. However, with further water restriction, genotype-specific differences were found to affect gene expression more than environmental conditions. These facts reflect a wide range of adaptation mechanisms in durum wheat under elevated [CO_2_] and water stress during vegetative growth, probably due to the complex regulatory network that takes place with both factors. Moreover, our study did not show a clear trend concerning the genetic advance in response to future climate change scenarios. Our results evidenced for durum wheat the need to take into account the genotypic variability for a greater understanding of plant adaptation to climate change. Moreover, the correlation network demonstrated that the combination of phenotyping and gene expression analysis is a useful approach to identify phenotype-genotype relationships and their behavior in response to different environments during vegetative stages.

## Author contributions

SM and JLA conceived and designed the experiments. SM, RV, and AA contributed to the experimental work. SM, RV, and JLA analyzed the data and interpreted the results. RV wrote the paper under the supervision of JLA, and SM and AA revised the manuscript. All authors have read and approved the final manuscript.

## Funding

This study was supported by the Spanish National Programme for Research Aimed at the Challenges of Society of the Ministry of Economy and Competitiveness (grants No. AGL2013-44147-R and AGL2016-76527-R). SM was the recipient of a fellowship “Presidente de la República PRONABEC-III” from Peruvian Government.

### Conflict of interest statement

The authors declare that the research was conducted in the absence of any commercial or financial relationships that could be construed as a potential conflict of interest.
