# Supplementary material for: Interactive Effects of Elevated [CO2] and Water Stress on Physiological Traits and Gene Expression during Vegetative Growth in Four Durum Wheat Genotypes
Source: Front Plant Sci. 2016 Nov 22;7:1738. doi: 10.3389/fpls.2016.01738 (PMC5118623; doi:10.3389/fpls.2016.01738)
Supplement: Supplementary file 1 [file Presentation1.PDF]

Supplementary Material

Interactive Effects of Elevated [CO_2] and Water Stress on Physiological Traits and Gene Expression during Vegetative Growth in Four Durum Wheat Genotypes

Susan Medina[1,2], Rubén Vicente[1*], Amaya Amador[3] and José Luis Araus[1]

[1]Integrative Crop Ecophysiology Group, Plant Physiology Section, Faculty of Biology, University of Barcelona, Barcelona, Spain

[2]Crop Physiology Laboratory, International Crops Research Institute for Semi-Arid Tropics (ICRISAT), Telangana, India

[3]Unitat de Genòmica, Centres Científics i Tecnològics, Universitat de Barcelona (CCiTUB), Barcelona, Spain

***Correspondence:** Rubén Vicente: vicenteperez.ruben@gmail.com

Supplementary Table S1. Primer pairs for durum wheat sequences used for qRT-PCR.

Acc. No.	Description	Symbol	Sequence	References
Ta2291	ADP-ribosylation factor	ADP-RF	(Fw) 5´-GCTCTCCAACAACATTGCCAAC-3´<br>(Rv) 5´-GCTTCTGCCTGTCACATACGC-3´	(Vicente et al., 2015b)
Ta2776	RNase L inhibitor-like protein	RLI	(Fw) 5´-CGATTCAGAGCAGCGTATTGTTG-3´<br>(Rv) 5´-AGTTGGTCGGGTCTCTTCTAAATG-3´	(Vicente et al., 2015b)
NC_021762	Rubisco large subunit, *rbcL* gene	RBCL	(Fw) 5´-ACGTGCTCTACGTTTGGAGG-3´<br>(Rv) 5´-CTTGGATACCATGAGGCGGG-3´	This study
AB020957	Rubisco small subunit, *rbcS* gene	RBCS	(Fw) 5´-AGCCTCAGCAGCGTCAGCAAT-3´<br>(Rv) 5´-CTTGGATACCATGAGGCGGG-3´	(Vicente et al., 2015b)
Y15897	Phosphoenolpyruvate carboxylase	PEPC	(Fw) 5´-TTGAGCAGAGCTTTGGGGAG-3´<br>(Rv) 5´-GGCTTTGGTGAAATGGGTGG-3´	This study
DQ124209	Cytosolic glutamine synthetase, *GS1a* gene	GS1	(Fw) 5´-AAGGACGGCGGGTTCAA-3´<br>(Rv) 5´-GCGATGTGCTCCTTGTGCTT-3´	(Yousfi et al., 2016)
DQ124212	Plastidial glutamine synthetase, *GS2a* gene	GS2	(Fw) 5´-GATGGAGGTTTCGACGTGAT-3´<br>(Rv) 5´-CAAGTCATGGCGAAGTGAAA-3´	This study
AJ890140	Dehydrin, *dhn11* gene	DHN11	(Rv) 5´-GAAGGAGGAGCACGAGGATG-3´<br>(Rv) 5´-CGTCACTAGACGAGCTGGAG-3´	This study
X78429	Dehydrin, *Td16* gene	DHN16	(Rv) 5´-AGATGCAGTAAAACTTCCCGAAAT-3´<br>(Rv) 5´-CCCGGGTACATACAAGCAGC-3´	(Ali-Benali et al., 2005)
D86327	Catalase-1, *cat1* gene	CAT	(Rv) 5´-TGACCGTTCCTCCGTTCGCCAT-3´<br>(Rv) 5´-TTGTCGTTGTTCCAGACGGGCG-3´	(Vicente et al., 2015b)
KP696754	Superoxide dismutase	SOD	(Rv) 5´-GGGTGTGGCTAGCTTTGGAT-3´<br>(Rv) 5´-TGCAGGTTTGACCCTTTGGT-3´	This study

Supplementary Table S2. Results of three-factor ANOVA for total leaf (LDW), shoot (SDW), root (RDW) and plant (PDW) dry weight, root/shoot ratio, number of tillers, chlorophyll content, stomatal conductance (g_s), N and C content, and N and C isotope composition ($\delta^{15}N$ and $\delta^{13}C$, respectively) in four durum wheat genotypes grown under ambient or elevated [CO_2] and optimal or restricted water supply. The values for least significant difference (LSD) are shown, and the symbols indicate statistical significance (*, *P* < 0.05; **, *P* < 0.01; ***, *P* < 0.001).

	CO_2 (C)	Water stress (W)	Genotype (G)	C × W	C × G	G × W	C × W × G
Moderate water stress (100% vs 60% pot capacity)							
LDW	0.34	0.34	0.48	0.48	0.68	0.68	0.96
SDW	0.80	0.80	1.13	1.13	1.60	1.60	2.26
RDW	**0.13*** **	0.13	0.19	0.19	0.27	0.27	0.38
PDW	0.79	0.79	1.12	1.12	1.58	1.58	2.24
Root/shoot	**0.09*** **	0.09	0.13	0.13	0.18	0.18	0.26
Tillers	1.4	1.4	2.0	2.0	2.8	2.8	4.0
Chlorophyll	**2.4***	2.4	3.3	3.3	4.7	4.7	6.6
g_s	56.3	**56.3****	79.7	**79.7***	112.6	112.6	159.3
N	**0.40****	0.40	0.57	0.57	0.80	0.80	1.14
$\delta^{15}N$	**0.34*** **	0.34	**0.49***	**0.49***	**0.69****	**0.69***	0.97
C	1.5	1.5	2.1	2.1	2.9	2.9	4.2
$\delta^{13}C$	**2.9*** **	2.9	4.1	4.1	5.7	5.7	8.1
Severe water stress (100% vs 30% pot capacity)							
LDW	**0.20*** **	**0.20*** **	0.29	**0.29*** **	0.41	0.41	0.57
SDW	**0.82*** **	**0.82****	**1.16****	**1.16*** **	1.64	1.64	2.32
RDW	**0.21*** **	**0.21*** **	**0.29*** **	**0.29****	0.41	0.41	0.58
PDW	**0.85*** **	**0.85*** **	1.20	**1.20*** **	1.70	1.70	2.40
Root/shoot	**0.12***	0.12	**0.17*** **	**0.17****	**0.24***	**0.24***	**0.34****
Tillers	**1.4***	**1.4*** **	1.9	**1.9***	**2.7***	2.7	3.9
NDVI	0.03	**0.03*** **	0.04	**0.04***	0.05	0.05	0.08
Chlorophyll	2.7	2.7	**3.8*** **	3.8	5.3	5.3	7.6
g_s	40.6	**40.6*** **	**57.5***	57.5	81.3	**81.3***	115.0
N	0.03	**0.03*** **	0.04	**0.04***	0.05	0.05	0.08
$\delta^{15}N$	0.29	0.29	0.41	0.41	0.58	0.58	0.83
C	**0.40***	0.40	0.57	0.57	0.81	0.81	1.14
$\delta^{13}C$	0.70	0.70	**0.99****	0.99	1.40	1.40	1.98

Supplementary Table S3. Pearson's correlation coefficients for physiological traits and transcript levels under different [CO_2], water supplies and sampling dates in four durum wheat genotypes. The symbols indicate a significant correlation between two parameters (*, *P* < 0.05; **, *P* < 0.01; ***, *P* < 0.001; ns, not significant). Abbreviations for the parameters are shown in the legends of Tables 1, 3 and S1.

	LDW	SDW	RDW	PDW	Root/shoot	Tillers	Chl	g_s	N	$\delta^{15}N$	C	RBCL	RBCS	PEPC	GS1	GS2	DHN11	DHN16	CAT	SOD
LDW		***	ns	***	**	***	***	ns	*	ns	ns	ns	ns	ns	ns	ns	ns	*	ns	ns
SDW	0.85		ns	***	**	**	**	ns	*	ns	ns	ns	ns	ns	ns	ns	ns	*	ns	ns
RDW	0.02	0.07		*	***	ns	*	*	*	ns	ns	*	*	ns	ns	**	*	*	ns	ns
PDW	0.78	0.93	0.43		ns	ns	***	*	ns	ns	ns	*	ns	ns	ns	ns	ns	**	ns	ns
Root/shoot	-0.47	-0.46	0.81	-0.12		**	ns	ns	**	ns	ns	ns	ns	ns	ns	ns	ns	ns	ns	ns
Tillers	0.67	0.49	-0.27	0.35	-0.45		ns	ns	*	**	ns	ns	ns	ns	ns	ns	ns	ns	ns	ns
Chl	0.60	0.53	0.40	0.63	-0.02	0.21		*	ns	ns	ns	*	*	ns	ns	**	ns	ns	*	ns
g_s	-0.28	-0.28	-0.38	-0.39	-0.18	0.23	-0.40		ns	ns	ns	ns	ns	ns	ns	ns	ns	ns	*	ns
N	0.42	0.41	-0.37	0.24	-0.54	0.36	0.25	-0.04		***	ns	ns	ns	ns	ns	ns	ns	ns	ns	ns
$\delta^{15}N$	-0.34	-0.32	0.13	-0.25	0.26	-0.46	-0.19	-0.17	-0.67		ns	ns	ns	ns	ns	ns	ns	ns	ns	ns
C	0.19	0.21	0.17	0.25	0.06	-0.04	0.15	-0.28	0.14	-0.12		ns	ns	ns	ns	ns	ns	ns	ns	*
RBCL	-0.16	-0.22	-0.44	-0.36	-0.27	0.09	-0.43	0.21	0.21	-0.34	-0.14		**	*	*	**	ns	ns	ns	ns
RBCS	0.13	0.03	-0.44	-0.14	-0.32	0.23	-0.42	-0.06	0.09	-0.07	0.07	0.56		ns	ns	***	*	ns	ns	ns
PEPC	0.08	0.01	0.02	0.02	-0.02	0.07	-0.06	-0.12	0.26	-0.29	0.15	0.39	0.28		*	ns	ns	ns	ns	ns
GS1	-0.15	-0.19	-0.05	-0.19	0.02	-0.05	0.00	0.30	0.13	-0.18	-0.15	0.36	0.06	0.42		ns	ns	ns	ns	ns
GS2	0.04	-0.02	-0.48	-0.19	-0.29	0.26	-0.54	0.00	0.05	-0.03	0.01	0.53	0.89	0.29	0.05		**	ns	ns	ns
DHN11	0.17	0.06	-0.40	-0.09	-0.34	0.29	-0.32	0.30	0.20	-0.19	-0.14	0.16	0.42	0.15	0.06	0.46		ns	ns	ns
DHN16	0.43	0.41	0.36	0.50	0.06	0.29	0.21	0.11	0.08	-0.33	-0.06	-0.15	-0.14	0.19	0.21	-0.13	0.08		ns	ns
CAT	-0.07	0.17	-0.26	0.06	-0.23	0.14	-0.42	0.38	0.32	-0.34	-0.07	0.27	0.19	0.20	0.20	0.21	0.34	0.10		ns
SOD	0.22	0.28	0.21	0.32	0.04	-0.10	0.14	-0.09	-0.24	0.20	0.36	-0.26	-0.05	-0.23	-0.21	-0.16	0.05	-0.03	0.11	

(A) Moderate water stress (100% vs 60% pot capacity)

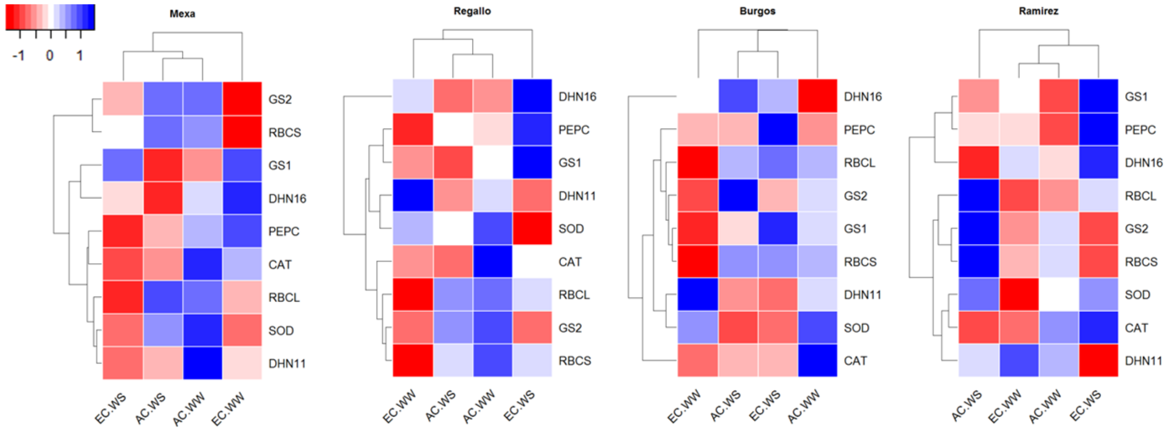

(B) Severe water stress (100% vs 30% pot capacity)

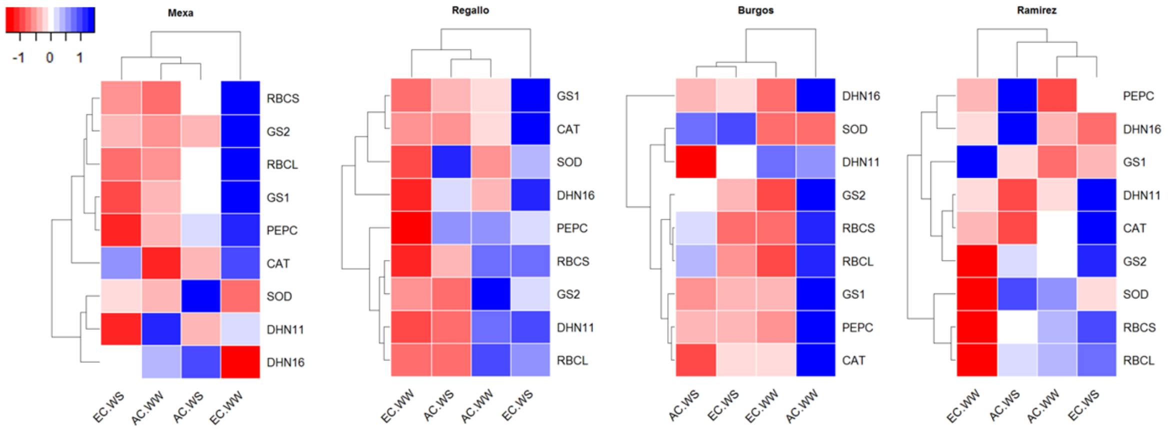

Supplementary Figure S1. Hierarchically clustered heat map of transcript changes in four durum wheat genotypes grown under ambient (AC) or elevated (EC) [CO_2] and well-watered (WW) or water stressed (WS) conditions: (A) moderate and (B) severe water stress. Data represent log_2 transformed fold changes of each treatment relative to the treatment under ambient [CO_2] and well-watered supply for each genotype. Values were scaled in the row direction as presented in the color key. RBCL, Rubisco large subunit; RBCS, Rubisco small subunit; PEPC, phosphoenolpyruvate carboxylase; GS1, cytosolic glutamine synthetase; GS2, plastidial glutamine synthetase; DHN11, dehydrin 11; DHN16, dehydrin 16; CAT, catalase; SOD, superoxide dismutase.
